# Prediction of Wall Heat Fluxes in a Rocket Engine with Conjugate Heat Transfer Based on Large-Eddy Simulation

**DOI:** 10.3390/e24020256

**Published:** 2022-02-09

**Authors:** Luc Potier, Florent Duchaine, Bénédicte Cuenot, Didier Saucereau, Julien Pichillou

**Affiliations:** 1 Centre Européen de Recherche et de Formation Avancée en Calcul Scientifique, 42 Avenue Gaspard Coriolis, 31057 Toulouse, France; lucpotier@gmail.com (L.P.); cuenot@cerfacs.fr (B.C.); 2 Centre National d’Etudes Spatiales, 52 rue Jacques Hillairet, 75612 Paris, France; julien.pichillou@cnes.fr; 3 ArianeGroup, Foret de Vernon, 27208 Vernon, France; didier.saucereau@ariane.group

**Keywords:** large-eddy simulation, conjugate heat transfer, rocket propulsion, cryogenic combustion

## Abstract

Although a lot of research and development has been done to understand and master the major physics involved in cryogenic rocket engines (combustion, feeding systems, heat transfer, stability, efficiency, etc.), the injection system and wall heat transfer remain critical issues due to complex physics, leading to atomization in the subcritical regime and the interactions of hot gases with walls. In such regimes, the fuel is usually injected through a coaxial annulus and triggers the atomization of the central liquid oxidizer jet. This type of injector is often referred to as air-assisted, or coaxial shear, injector, and has been extensively studied experimentally. Including such injection in numerical simulations requires specific models as simulating the atomization process is still out of reach in practical industrial systems. The effect of the injection model on the flame stabilization process and thus on wall heat fluxes is of critical importance when it comes to the design of wall-cooling systems. Indeed, maximizing the heat flux extracted from the chamber can lead to serious gain for the cooling and feeding systems for expander-type feeding cycles where the thermal energy absorbed by the coolant is converted into kinetic energy to drive the turbo-pumps of the feeding system. The methodology proposed in this work to numerically predict the flame topology and associated heat fluxes is based on state-of-the-art methods for turbulent reactive flow field predictions for rocket engines, including liquid injection, combustion model, and wall treatment. For this purpose, high-fidelity Large Eddy Simulation Conjugate Heat Transfer, along with a reduced kinetic mechanism for the prediction of $H_{2}/O_{2}$ chemistry, liquid injection model *LOx* sprays, and the use of a specific wall modeling to correctly predict heat flux for large temperature ratio between the bulk flow and the chamber walls, is used. A smooth and a longitudinally ribbed combustor configuration from JAXA are simulated. The coupling strategy ensures a rapid convergence for a limited additional cost compared to a fluid-only simulation, and the wall heat fluxes display a healthy trend compared to the experimental measurements. An increase of heat transfer coherent with the literature is observed when walls are equipped with ribs, compared to smooth walls. The heat transfer enhancement of the ribbed configuration with respect to the smooth walls is coherent with results from the literature, with an increase of around +80% of wall heat flux extracted for the same chamber diameter.

## 1. Introduction

Heat flux management is essential in the design of a cryogenic combustion chamber [1]. The cooling system evacuates the heat produced by cryogenic fuel combustion at high pressure and ensures the engine integrity. This is a common way of cooling cryogenic chamber walls since the conception of the *V2* relies on the use of cooling channels integrated in the combustion chamber liners and fed with the fuel stored at very low temperature. For critical parts like a nozzle throat, a film cooling is sometimes employed [2]. Moreover, high chamber pressure, which allows one to reduce engine size and weight, requires maximum heat extraction. In the case of expander-type cycle feeding system, the increase in heat extracted also maximizes the turbomachinery efficiency and then the thrust. Maximizing heat fluxes has therefore become a prime design criterion.

One simple way to increase heat transfer to the coolant is to increase the chamber wall surface, either in length or in diameter, but this goes against size and load limitations. Heat enhancement techniques that do not compromise the engine compacity are then required. To increase the exchange area exposed to burnt gases without increasing the engine size, ribbed wall surfaces can be used.

The main effect of ribs is to increase the exchange surface. Depending on the geometry, this can reach +80% in a thrust chamber [3]. This process has been explored in heat exchangers, blade cooling [4,5,6], or chemical processes [7,8], such as thermal cracking in the petroleum industry [9]. A lot of rib geometries exist, such as squared, round, triangular, or trapezoidal. The installation can be longitudinal, transverse, or helical, with various dimensions. All these parameters have an important impact on performances in terms of pressure losses and heat transfer enhancement with regard to an equivalent smooth surface.

Ribs used in rocket combustors are positioned in the direction of the flow for stability reasons. Indeed, helical and transverse ribs may induce rotational motion, which may destabilize the rocket. The Orbit Transfer Rocket Engine Technology Program initiated by NASA (National Aeronautics and Space Administration) in the 1990’s was the first to explore heat flux enhancement thanks to ribbed walls [3]. A 2D preliminary study first showed that a rib height of 0.040 inches was optimum in terms of heat flux enhancement (42% increase compared to smooth walls). Then this was tested in a dual experiment comparing an $H_{2}/O_{2}$ calorimeter chamber, with smooth walls and with 0.040 inches ribs. Two operating pressures were targeted (850 psia and 1050 psia), and mixture ratio varied from 5.0 and 7.0. The heat flux enhancement was found to be of the same order as in the 2D experiment (+50% at $P_{ch}=850$ psia, +40% at $P_{ch}=1050$ psia). It was also found that the heat flux enhancement is very sensitive to mixture ratio and chamber pressure: higher mixture ratio and chamber pressure increase the efficiency of the ribbed wall.

The JAXA (Japan Aerospace Exploration Agency) also experimentally studied heat flux enhancement using ribbed walls [10,11] with two experimental thrust chambers: one with smooth walls and the other with ribbed walls. The two chambers have been designed to operate with $LOx/GH_{2}$ but only at one targeted pressure and mixture ratio. They obtained significant heat flux enhancement as expected, with similar rib efficiency to the one obtained in the NASA experiments. It was also observed that the small size of the ribs (h≈1 mm) does not change the macroscopic flow because the ribs’ height is below the boundary layer thickness.

Ribbed walls were also studied numerically. JAXA published a RANS study of their two experimental thrust chambers [10]. Betti et al. [12] numerically reproduced the ribbed NASA experiment [3] with a RANS approach. As the measurement was made in the cooling channel along the chamber, the RANS simulation of the cold flow was coupled to a 1D model for conduction in the liner and the cooling channel. The results showed good agreement with the heat flux enhancement measured in the experiment. The second part of the study was dedicated to the evaluation of rib efficiency in a $LOx/CH_{4}$ ribbed burner, equivalent to the one of JAXA [10]. Analysis of the flow fields emphasized the importance of stratification in the inter-rib space and its effect on heat flux.

Cryogenic combustion involves many complex phenomena: fast and highly exothermic $H_{2}/O_{2}$ kinetics, high operating pressure, highly turbulent flow and mixing, and strong heat exchange [13]. Large Eddy Simulation (LES) allows one to describe most of these phenomena and their interaction in an unsteady context producing better results than RANS approaches. The objective of this paper is to investigate numerically, thanks to LES, the heat transfer enhancement on the JAXA ribbed configurations compared to the smooth case [10,11] with state-of-the-art methods for turbulent reactive flows including liquid injection, combustion kinetics, wall treatment, and Conjugate Heat Transfer (CHT). The LES code AVBP (Advanced Virtual Burner Program) developed by CERFACS has already proven its ability to reproduce combustion in representative rocket engine conditions [14,15,16,17,18,19] in transcritical and supercritical regimes as well as for subcritical regimes [20] and for transient ignition [21]. The CHT approach is used to impose the thermal boundary on the combustion chamber wall further away from the region of interest where the thermal measurements have been performed and to take into account the dependency of the heat fluxes on the wall temperature. Although measurements are available for comparisons, regarding all the uncertainties at the experimental and numerical levels, the objective of the study is not to perfectly match the experimental measurements but to capture the global trends without any fitting of the LES models, as is generally done when using lower-order turbulence modeling such as RANS solvers.

The paper is organized as follows. First, the smooth and ribbed JAXA combustion chamber configurations are described. Then, the computational setup is presented focusing on the numerical approach to solve the reactive flow fields, the two-phase flow approach including the liquid injection model, the combustion modeling, the wall treatment, and the CHT approach. Finally, the results are analyzed in terms of flow and combustion dynamics, and then the heat transfers are described.

## 2. The JAXA Chamber Configurations

**Figure 1:**
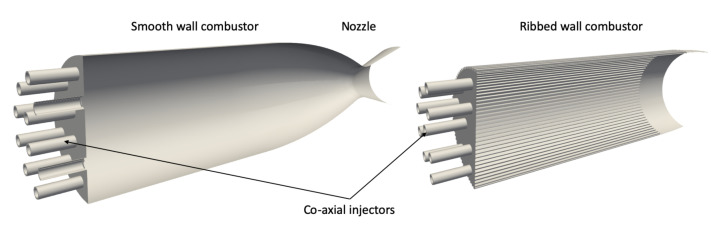
The smooth (**left**) and ribbed (**right**) calorimeter chambers of JAXA [11].

The JAXA experiment includes two equivalent calorimeter chambers: one with smooth walls and one with ribbed walls shown in Figure 1. The smooth and the ribbed chambers have the same overall geometrical characteristics, with only a difference on the cylindrical part (liner), which is longer in the ribbed case. The nozzle is the same in the two configurations. The main geometrical features are summarized in Table 1. Other details about geometry and experiments can be found in [11]. Chamber walls in the cylindrical section are about 1 cm thick. Ribs are 1 mm high and 1.23 mm wide. Ninety ribs are equally placed along the circumference of the chamber, leading to an inter-rib distance of 1 mm.

Both chambers were equipped with a set of 18 *LOx*/$GH_{2}$ coaxial injectors, with the central injector positioned with a recess of r=1 mm from the injection faceplate. Large injector lips favor the flame stabilization. They operate at approximately same pressure (*P* = 35–36 bars) and oxygen/fuel ratio (MR = 5.5–5.6). The operating conditions studied here are given in Table 2.

A water cooling system is used to reach steady conditions. Details about the cooling system have not been published, but it is made of water flowing at 350 K inside circumferential channels positioned all along the chamber.

## 3. Computational Setup

The thermally coupled simulations have been performed with the in-house solvers AVBP (Advanced Virtual Burner Program) for the fluid and AVTP (Advanced Virtual Thermal Program) for the solid. This section describes the solvers and modeling strategies used. The injection of gaseous hydrogen and liquid oxygen as well as the combustion modeling are of primary importance when it comes to the prediction of the flame position and thus on wall-heat fluxes.

### 3.1. Numerical Approach to Solve the Reactive Flow Field

#### 3.1.1. Numerical Scheme

The parallel LES code, AVBP [16,22,23], solves the full compressible Navier–Stokes equations using a two-step time-explicit Taylor–Galerkin scheme (TTG4A) for the hyperbolic terms based on a cell-vertex formulation [24], along with a second-order Galerkin scheme for diffusion [25]. TTG4A provides high spectral resolution and low numerical dissipation and dispersion, which is adequate for LES [26]. This scheme provides third-order accuracy in space and fourth-order accuracy in time. Such numerics are especially designed for LES on hybrid meshes and have been extensively validated in the context of turbulent reacting flow applications [27,28,29,30]. The time step is controlled by the acoustic CFL number (0.7 for the present computations). The unstructured hybrid approach enables refinement of the mesh in zones of interest by using prisms in the wall region [31,32]. The unresolved Sub-Grid Scale (SGS) stress tensor is modeled using the Boussinesq assumption [33], and the SGS viscosity $\mu_{SGS}$ is computed with the SIGMA model [34]. The SGS heat flux is modeled using the classical gradient-diffusion hypothesis [33] that relates the SGS heat flux to the filtered temperature gradient using a SGS thermal conductivity. This approach postulates a direct analogy between the momentum and heat transfer through the SGS turbulent Prandtl number ($Pr_{SGS}=\mu_{SGS}\;C_{p}\left(T\right)/\lambda_{SGS}$), here fixed at $Pr_{SGS}=0.5$.

The liquid phase is computed with an Euler–Euler approach describing the liquid as a continuous medium corresponding to spray statistics [35]. The Eulerian field is locally mono-disperse in each computational cell. The same numerical scheme used for the gas is applied for the dispersed phase. The evaporation model for liquid phase is the Abramzon–Sirignano model [36].

#### 3.1.2. Liquid Injection Model

Coaxial injectors are used in rocket engine for their efficiency in the atomization of the liquid jet. Atomization is a very complex phenomenon that is not yet fully understood and modeled. The pioneer works of Farago and Chigier [37] and Rehab [38], and the more recent studies of Lasheras, Hopfinger and Marmotant [39,40,41], have produced some insight into the different phases of this process. The breakup of a liquid jet by a fast gas stream may exhibit different regimes that can be classified using three non-dimensional numbers: the Weber number We, the liquid Reynolds number $Re_{l}$, and the momentum ratio *J*:

(1) $$\begin{array}{cccccc} We & =\frac{\rho_{g}\Delta_{U}^{2}D_{l}}{\sigma } & \;Re_{l} & =\frac{U_{l}D_{l}}{\nu_{l}} & \;J & =\frac{\rho_{g}U_{g}^{2}}{\rho_{l}U_{l}^{2}} \end{array}$$

where $\rho_{g}$ and $\rho_{l}$ are the density of the gas and liquid, $\Delta u=u_{g}-u_{l}$ is the relative velocity between the gas and the liquide phase, $D_{l}$ is the liquid injection diameter, σ is the surface tension, and $\nu_{l}$ is the liquid kinematic viscosity. The Weber number represents the ratio of aerodynamic destabilizing forces over the stabilizing surface tension forces.

**Figure 2:**
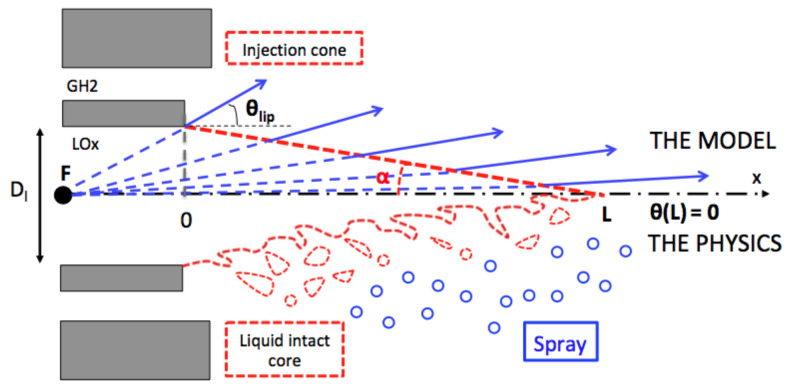
Atomization process (**bottom** half) and liquid droplet injection model (**top** half).

Both smooth and ribbed configurations have similar injection characteristics because the injectors are the same and the operating conditions are very similar. The liquid Reynolds number $Re_{l}$ and the Weber number We are high, which, following the classification of Lasheras [39], leads to a liquid jet atomization of fiber type. As the direct simulation of the atomization is still the subject of intense research and requires computational resources that are not compatible with LES of a rocket engine, a phenomenological injection model is used (Figure 2). This “focal-point” model was introduced introduced by Potier et al. [42,43] to inject an already atomized spray, as illustrated in Figure 2 (top). The liquid potential core not influenced by the surrounding flow is not computed by the CFD solver and is replaced by a conical surface of length *L*. Particles of diameter $d_{l}$ are injected uniformly along this surface with a varying angle starting at $\theta_{lip}$ at the lip and going to 0 at the tip of the cone. The injection mean axial velocity corresponds to the flow rate of the LOx stream. Considering the operating conditions of both chambers Table 2, the injection parameters have been established for the two configurations and are reported in Table 3. The liquid intact core length *L* has been fixed with the correlation of Villermaux [44]. This may lead to a different flame length but has little effect on the wall heat flux [42]. The injection diameter is evaluated with the correlation of Lasheras [39]. The injection is carried out on a short conical surface approximately ten times the section area of the oxygen injector, and the liquid volume fraction imposed at the oxygen inlet is high: $\alpha_{l}\approx 0.1$. The droplet Stokes number St is very low, indicating that particles behave like tracers and follow the carrying phase motion, including turbulent fluctuations.

#### 3.1.3. Kinetics for the Combustion Model

**Figure 3:**
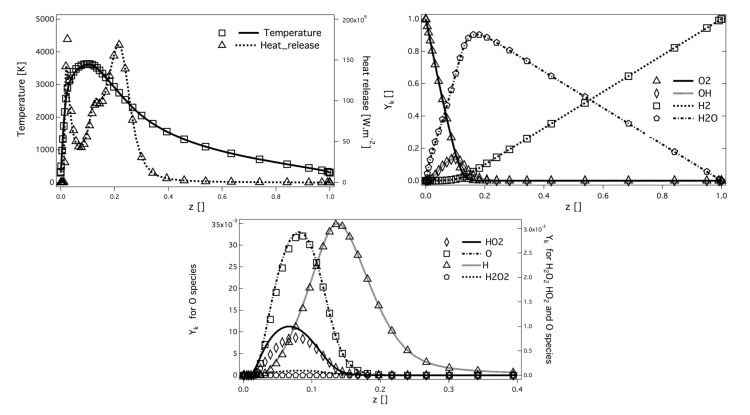
Laminar strained diffusion flame (a=2000 s${}^{-1}$) at 40 bar. Profiles of temperature and heat release, main species mass fraction, and intermediate species mass fraction, for *H2-O2-12S* scheme [45] (symbols) and *Sandiego* mechanism (lines) [46].

The chemical scheme used in this study for $H_{2}/O_{2}$ combustion is the 8-species, 12-reactions reduced scheme of Boivin [45], called H2-O2-12S in the following. This scheme has been compared to the San Diego detailed scheme [46] on academic flames generated with the kinetic solver CANTERA [47]. The reduced scheme exhibits very good behavior for laminar premixed flames. The laminar flame velocity $s_{L}$, the laminar flame thickness $\delta_{L}$, and the burnt gases temperature are well recovered on a wide range of $O_{2}/H_{2}$ mass ratios at ambient pressures and up to 100 bar [45]. To be consistent with rocket engine applications, where flames are mostly pure diffusion flames, the reduced scheme has been validated over 1D non-premixed counterflow flames, and showed good agreement with the reference *Sandiego* mechanism, as observed in Figure 3. The response to strain rate for ambient pressure and up to 40 bar has shown very good prediction of flame thickness, maximum temperature, and levels of heat release.

Finally, two-phase jet flames have been computed in [42] with the present kinetic scheme. The structure of the turbulent diffusion flames was well recovered, and results confirm that it is driven by evaporation.

With a CFL imposed to 0.7, the resulting time-step is of the order of $\Delta t=0.5\times 10^{-8}$ s. Due to the high reactivity of $H_{2}/O_{2}$ mixtures, the *H2-O2-12S* scheme exhibits very small characteristic time scales, and sub-cycling is required for chemistry integration (≈30 sub-cycling iterations). Thanks to a refined mesh, turbulence-chemistry interaction is fully resolved here. Indeed, in the flame zone the maximum mesh size is 0.1 mm, i.e., which is the order of magnitude of a non-premixed $H_{2}/O_{2}$ strained laminar flame thickness. To capture flame anchoring on the lip, the minimal cell size has to be ≈50 µm.

### 3.2. Numerical Approach to Solve the Heat Equation in the Solid

The heat conduction solver AVTP [48] solves the heat transfer equations in solids:

(2) $$\rho_{s}C_{s}\frac{\partial T(x,t)}{\partial t}=-\frac{\partial q_{i}}{\partial x_{i}}$$

where *T* is the temperature, $\rho_{s}$ the density, and $C_{s}$ the heat capacity in the solid. The heat flux *q* follows Fourier’s law:

(3) $$q_{i}=-\lambda_{s}\frac{\partial T}{\partial x_{i}}$$

where $\lambda_{s}$ is the conductivity of the solid. The capacity and conductivity are tabulated with temperature. Based on the AVBP data structure, the parallel conduction solver uses a second-order Galerkin diffusion scheme [25]. Time integration is done in this study with an explicit first-order forward Euler scheme. The time-step is imposed by the Fourier number $F=\frac{a_{s}\Delta t}{\Delta x^{2}}=0.1$, where Δx is the minimum cell size and $a_{s}=\frac{\lambda_{s}}{\rho_{s}C_{s}}$ is the thermal diffusivity.

Material used for solid heat conduction is a copper alloy for the liner and stainless steel for the injection plates and injectors lips. Their mean characteristics are given in Table 4. The heat capacity $C_{s}$ and the thermal conductivity $\lambda_{s}$ follow an evolution whose temperature is not given here, for confidentiality reasons (for the copper alloy especially).

### 3.3. Conjugate Heat Transfer Coupling Approach

The Conjugate Heat Transfer (CHT) problem is solved with two independent solvers that exchange information. In the CHT problem, information is exchanged only at physical boundaries. The coupled application is generated thanks to the CWIPI library [48,49], which manages the communications between the LES solver AVBP and the conduction solver AVTP.

**Figure 4:**
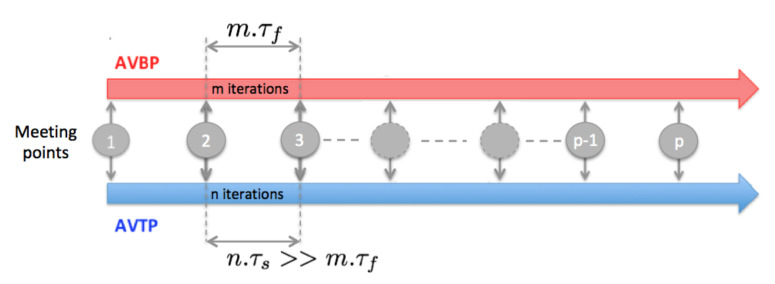
Coupling strategy to reach thermal steady state in the solid.

The characteristic flow time expressed as the convection time $\tau_{f}$ is of the order of few ms, while the characteristic time for conduction in the combustors liner given by

(4) $$\tau_{s}=\frac{L_{s}^{2}}{a_{s}}$$

is generally of the order of few *s* ($L_{s}$ is the characteristic thickness of the liner here). This is problematic if one wants to compute transient states. However, in the present study, only the steady state is targeted and the coupling strategy developed at CERFACS [50,51] to rapidly reach steady state that is used. This method has been used with success in numerous applications [32,52,53,54,55,56,57,58,59]. It consists in desynchronizing the LES and conduction solvers, each advancing with its own time-step adapted to its physics (CFL-driven for reactive flow, Fourier-driven for solid conduction). The coupled simulation is started with an initial guess of the solid and fluid solution, and after a transient phase the steady state is reached. Information is exchanged at a given frequency *f*. At each *meeting point* (Figure 4), the temperature calculated with AVTP is imposed as a wall boundary condition in AVBP, while the heat flux evaluated in AVBP is imposed as boundary condition for AVTP. Between two *meeting points*, the flow is advanced in time by $n\tau_{f}$, while the solid is advanced in time by $m\tau_{s}$.

This procedure is equivalent to lower the solid heat capacity while conserving the same conductivity. Indeed, going back to the conduction equation for a simple heat transfer problem, the steady state is reached when the partial derivative of the temperature with respect to time is null:

(5) $$\rho_{s}C_{s}\left(x,t\right)\frac{\partial T(x,t)}{\partial t}=\frac{\partial }{\partial x_{i}}\left(\lambda_{s}\left(x,t\right)\frac{\partial T(x,t)}{\partial x_{i}}\right)$$

As a result, a modification of the density $\rho_{s}$ or the heat capacity $C_{s}$ of the solid in Equation (5) does not change the steady state solution, only the characteristic time-scale of the solid $\tau_{s}$ (Equation (4)). As a consequence, decreasing $C_{s}$ makes the two solvers’ timescales compatible. However, this method is only valid to reach thermal steady state and cannot be used to investigate temporal temperature evolutions in the solid. Indeed, an increase in the heat diffusivity through the decrease of $\rho_{s}$ or $C_{s}$ increases the thermal activity ratio and the penetration of the temperature fluctuation in the solid.

In this study, the characteristic flow time is evaluated as ten convective time $\tau_{f}=10\;\;\frac{L_{f}}{{\bar{u}}_{f}}$, where ${\bar{u}}_{f}$ is the mean velocity of the burnt gases and $L_{f}$ is the combustion chamber length. For the simplified diffusion problem described by the heat equation (Equation (2)) with simple boundary conditions, the time-scale $\tau_{s}$ represents the time needed to reach about 60% of the final temperature, while $5\tau_{s}$ is needed to reach 99% of the final value. The simulation time for the liner is then taken above $5\tau_{s}$.

### 3.4. Meshes and Boundary Conditions

**Figure 5:**
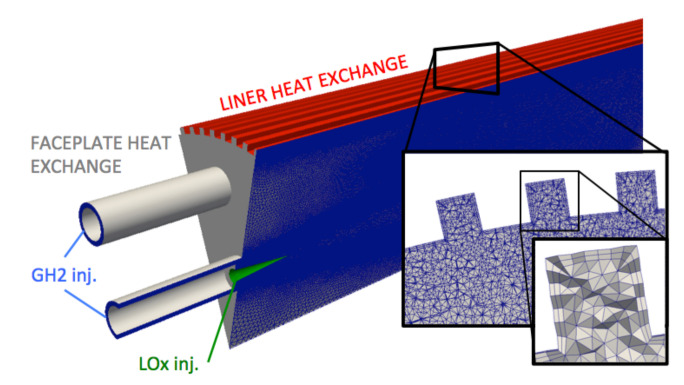
Fluid mesh for JAXA calculation. Prism aspect ratio is kept under five. There are only two stacks of prisms to limit cell deformation.

The fluid computational domain corresponds to a 30 degree sector containing an outer injector and half of an internal injector. Symmetries are applied on both lateral sides of the numerical domain. In addition, the nozzle has been truncated and the chamber pressure is imposed at the outlet of the domain. A prism layer is placed along the ribbed walls, limited to two stacks of prisms. Particular attention has been paid to concave corners, where a “pull-back” ratio has been imposed to limit the deformations of the prisms in the corner. Ribs are resolved with ≈8 cells in the rib height and width (see Figure 5). This leads to wall resolution $y^{+}\approx >50$, which calls for the use of the wall modeling approach. As boundary layers are not resolved, wall fluxes are evaluated with a coupled wall law specifically developed for high-temperature ratios between the wall and the bulk flow [60]. In this law-of-the-wall formulation, the energy and momentum equations are coupled for large temperature variations (i.e., large density variations). The details and validations of this wall modeling are described in [60] for smooth walls and in [43] for smooth and ribbed walls. All liners are treated with this wall model with a temperature obtained by the thermal solver during the coupling. The GH2 injections are treated as inlets with imposed mass-flow rates and temperature with an NSCBC treatment [61]. Pressure is imposed at the outlet of the computational domain with a NSCBC formalism. The conical liquid injection surface is treated as an adiabatic wall-slip condition.

**Figure 6:**
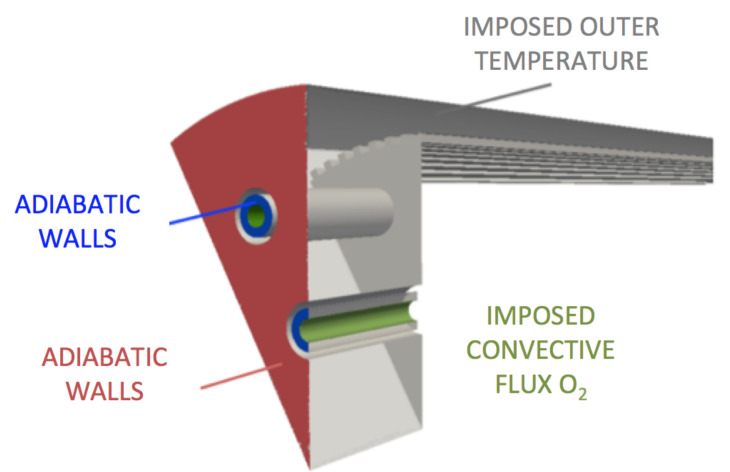
AVTP solid domain boundary conditions.

The solid computational domain is composed of the liner, the injection plate, and the lips of the injectors (Figure 6). The associated mesh is fully composed of tetrahedrons. At least 10 points have been placed in the liner thickness and in the ribs’ height and width. The surface interfacing with the fluid is mapped with triangles with the same characteristic size as the fluid mesh, to avoid interpolation errors. Resulting meshes size are reported in Table 5. For solid boundary patches interfacing with the fluid, a Neumann boundary condition to impose heat flux is used. The other boundary conditions of the solid domain are illustrated on Figure 6: on the outer wall of the liner, the water temperature 350 K is direcly imposed. The back of the injector faceplate and the back of injectors lips are considered adiabatic. A convective heat flux is imposed in the *LOx* injection system:

(6) $$\Phi_{Lox}=h_{LOx}\left(T_{LOx}-T_{w}\right)$$

with $T_{LOx}$ the *LOx* injection temperature, $T_{w}$ the wall temperature computed by the solid solver, and $h_{LOx}$ a convective coefficient evaluated on the inner side of the oxygen injector thanks to the empirical Colburn correlation [62]:

(7) $$h=0.023\;Re_{f,D_{h}}^{0.8}Pr^{1/3}\;\lambda /D$$

where *D* is taken as the diameter of the *LOx* injector and all properties of the fluid have been evaluated at an estimated film temperature $T_{film}=185$ K, considering that wall temperature does not exceed the temperature of the $H_{2}$ injection (270 K).

## 4. Results

The cost of each simulation was about 300,000 CPU hours for two convective times on a 35-million-cell mesh. The presentation of the results is divided into three parts. First, the flow structure is discussed based on the ribbed configuration solution (the flow topology of the smooth configuration is very similar). Then, flame shape and structure are investigated as well as the flame anchoring mechanism and its interaction with the walls. Heat flux distribution obtained from the resolution of the CHT problem is analyzed, and the temperature fields of the solid liner and the ribs efficiency are also studied. Finally, the mean heat flux profiles are compared to the measurement in both ribbed and smooth configurations.

**Figure 7:**
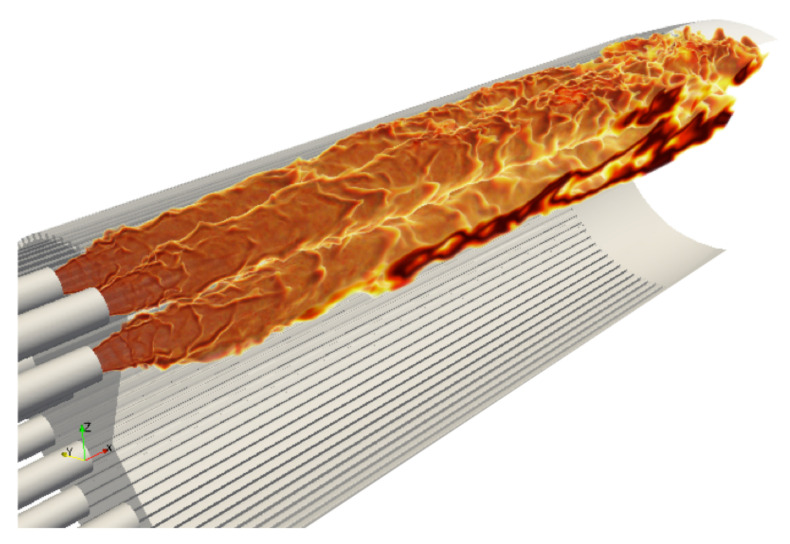
Multiple iso-surfaces of temperature (from 3000 to 3600 K); transparency depends on temperature. The $30^{\circ }$ sector simulated is duplicated to represent three injectors flames.

**Figure 8:**
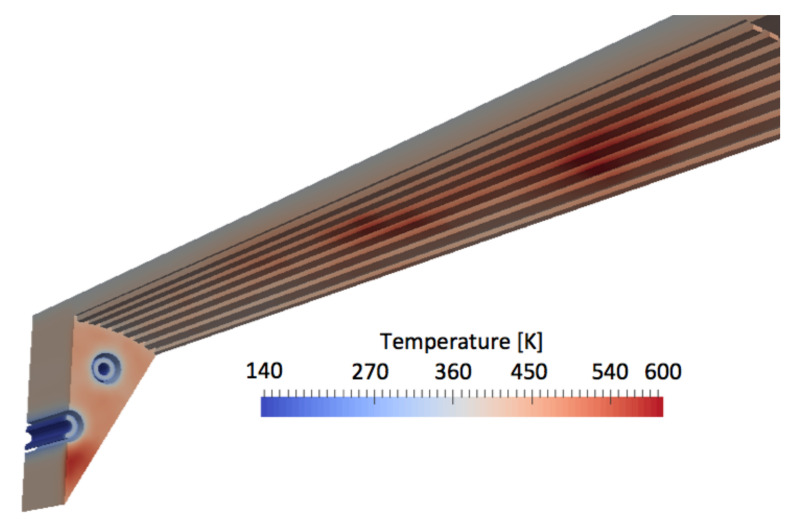
Converged thermal solution in the ribbed liner.

To first illustrate the global topology of the flow, a converged instantaneous partial view of the flames is shown Figure 7. The flame is wrinkled near the injection due to the shear imposed by the coaxial $H_{2}$ jet. The most turbulent part of the flame is located further downstream in the chamber when it encounters the ribbed walls. The flames appear to be very long, with their lengths being almost equivalent to the liner length (until the convergent part of the nozzle) for both configurations. The converged thermal solution in the solid (Figure 8) shows that the high temperature region at the center of the faceplate is due to a large recirculation of burnt gases induced by the absence of central injector in this configuration.

### 4.1. Description of the Two-Phase Flow

**Figure 9:**
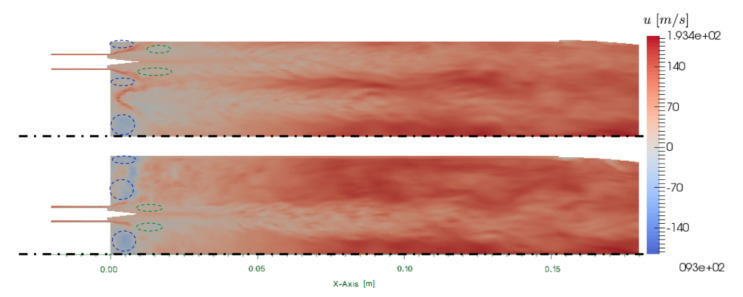
Ribbed JAXA chamber: cuts of mean axial velocity *u* at $0^{\circ }$ (**bottom**) and $15^{\circ }$ (**top**). Recirculation zones are evidenced by the dashed line circles.

In this section, only the results of the ribbed chamber simulation are described because the general structure of the flow is very similar in the smooth chamber. To describe the topology of the flow, an axial cut crossing the center of the inner injector is used (Figure 9). A second cut shifted by $15^{\circ }$ to cross the center of the outer injector is also shown. The mean flow fields discussed here have been obtained by averaging the simulation over two convective times after the convergence of the CHT problem. The mean axial velocity fields show a strong deviation of the hydrogen jet due to the liquid phase evaporation and the expansion of the burnt gases. Evidenced with dashed blue lines, short corner recirculation zones are observed on the side of the injectors and near the side walls. A second type of recirculation zone (in green dashed line) is observed in the jet, due to the over-expansion of the hydrogen jet provoked by the short intact core length. This phenomenon would not be present anymore with a longer injection cone. The cut in the inner injector plane is aligned with a micro-channel at the ribbed wall where lower axial velocity is observed. The axial velocity is importantly reduced in the micro-channel due to higher friction. On the contrary, the cut of the outer injector is aligned with the top of a rib and axial velocity is more important close to the wall. At the end of the chamber, the axial velocity field tends to homogenize but lower velocity traces of the evaporating oxygen stream are still present.

**Figure 10:**
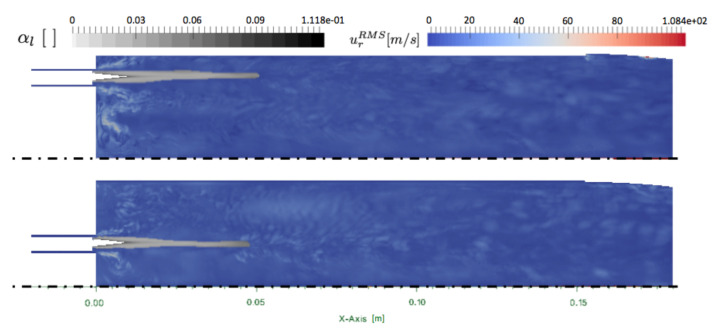
Ribbed JAXA chamber: cuts of radial RMS velocity $u_{r}^{RMS}$ with superimposed field of liquid volume fraction $\alpha_{l}$ at $0^{\circ }$ (**bottom**) and $15^{\circ }$ (**top**).

The turbulent activity is observed with the radial RMS velocity in Figure 10. The RMS radial velocity is low in the oxygen jet where the liquid phase evaporates. The liquid volume fraction shows a quite highly laden spray ($\alpha_{l}=0.118$) at the injection surface, which rapidly decreases under 0.03. The length of the spray is about 5 cm long, with the inner and outer jets being almost equivalent. The liquid jet is very stable since it is not perturbed by the turbulent activity.

### 4.2. Flame Structure

**Figure 11:**
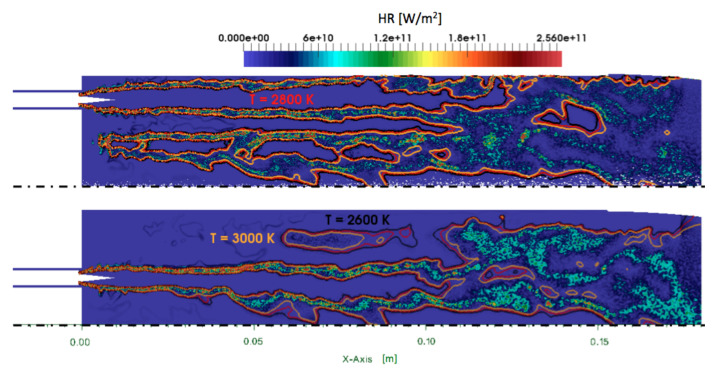
Ribbed chamber: instantaneous cuts of heat release HR with superimposed iso-lines of temperature.

The flame shape is evidenced in Figure 11 with instantaneous heat release fields. The flames occupy almost all the simulated domain and are about 15 cm long. In this configuration, the flame is anchored at the injector lip, and no lift-off is observed. The deviation of the inner flame toward the axis of the chamber is attributed to the absence of injector at the center of the faceplate and the large recirculation zone installed there. However, the center and outer flames are very similar. In the following, only the outer flame is analyzed.

**Figure 12:**
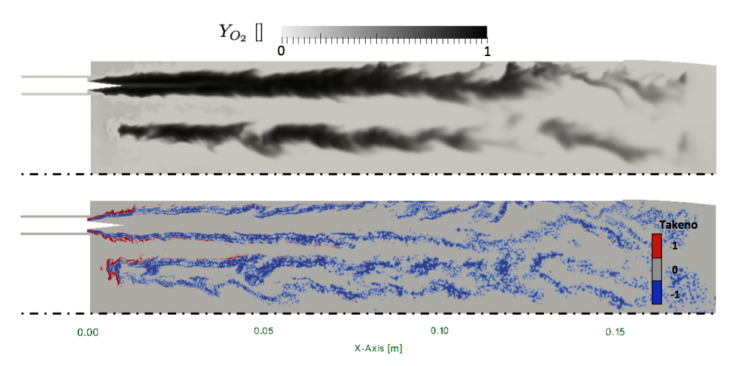
Ribbed JAXA chamber instantaneous cut of the outer injector. (**Top**): oxygen mass fraction $Y_{O_{2}}$; (**bottom**): Takeno index, [−1]: non-premixed flame, and [1] premixed flame.

**Figure 13:**
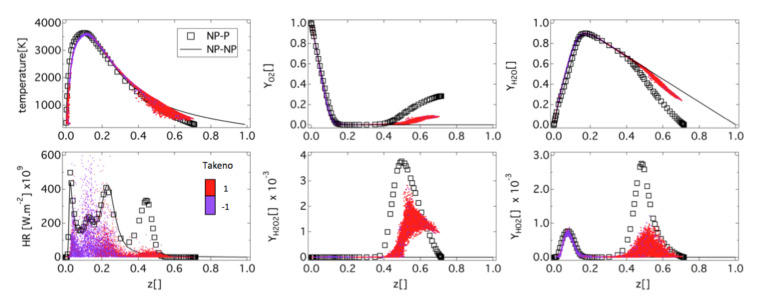
Scatter plots of heat release, temperature, and species mass fraction, plotted along the mixture fraction *z* colored by Takeno index, compared to 1D pure diffusion flame (NP-NP) and mixed Non Premixed-Premixed (NP-P) flame (a=5000 s${}^{-1}$).

**Figure 14:**
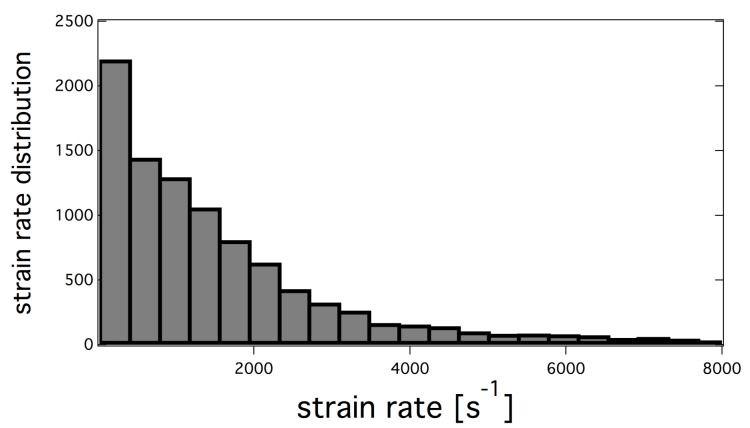
Ribbed JAXA chamber strain rate distribution taken along the stoichiometric iso-surface (for $z=z_{st}$).

Despite the fact that the flame is well anchored on the injector lip, still a small amount of the oxygen spray evaporates in the hydrogen jet as observed on Figure 12 (top). Consequently, the flame structure is a non purely diffusion flame type, as evidenced by the instantaneous field of Takeno index in Figure 12 (bottom). However, the structure of the flame tends rapidly toward a pure diffusion flame after x=2 cm. In fact, large injector lips allow for strong anchoring of the flame limiting the effect of the injection model on the flame structure. To confirm the flame structure, scatter plots are built from the solution close to the injector. The scatter plots of the flame structure are presented in Figure 13. Points are colored with the Takeno index. The two 1D strained laminar flame non-premixed and premixed flames are added for comparisons. A first observation is the dispersion of the heat release in comparison with the laminar flame profiles. This is linked to the distribution of turbulent strain rates shown on Figure 14, which has a strong impact on diffusion flames. Heat-release scatter plots follow the pure diffusion flame structure for low values of mixture fraction z but tend to recover Non Premix-Premix (NP-P) profiles at higher values of z. The third peak of heat release observed in the NP-P reference laminar flame is very low in the simulation. The scatterplots of $HO_{2}$ and $H_{2}O_{2}$ species mass fraction also exhibit lower production of these radicals in the premixed region of the flame.

### 4.3. Comparison between Ribbed and Smooth Chambers

In this section, a comparison is made between the ribbed and smooth chambers. The influence of the ribbed wall on both the topology of the flow and the conduction in the solid liner is investigated. Averaged solutions are used for the discussion, corresponding to two convective times of the fluid domain. Solid solutions for the liners of the two burners have been averaged according to one characteristic conduction time. Bottom of the ribbed surface designates the most external face of the micro-channels, whereas top of the rib will designate the face of the ribbed exposed to the bulk flow.

**Figure 15:**
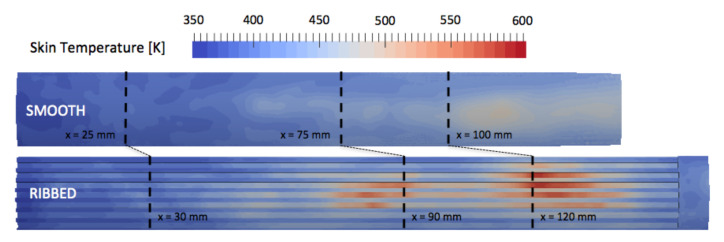
Time-average temperature of the inner wall of the solid liner for JAXA smooth (**top**) and ribbed (**bottom**) burners extracted from AVTP averaged solutions.

The averaged inner wall temperature fields are extracted from the solid solution and are represented in Figure 15 for the ribbed and smooth coupled simulations. It is recalled that the smooth and ribbed chamber lengths differ, and the investigated axial planes will be positioned at the same percentage of the respective lengths. This choice is motivated by the fact that in both configurations, the flames extend from the injector to the end of the combustion chamber. Temperature for the ribbed burner is significantly higher at the top of the ribs, whereas the bottom of the ribs are exposed to temperatures of the same order as in the smooth configuration. As observed previously, the flames in both configurations almost reach the end of the combustion chamber. The averaged temperature fields of liner exhibit similar pattern for the two chambers: two high-temperature spots are observed along the axial direction, with the first temperature spot being positioned around 1/2th of the chamber lengths and the second around 2/3th of the chamber lengths. The shape and location of these high-temperature regions are linked to the flame shape (mainly annular due to the injection system) and interactions between the external flame and the wall.

**Figure 16:**
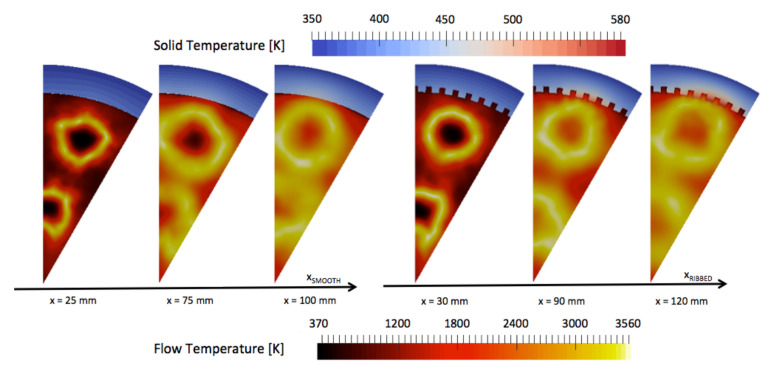
Time-averaged temperature on cross-section cuts in fluid and solid domains for the smooth and ribbed configurations.

Three cross-sectional cuts are used in support of the observation made on Figure 15. The first cut is used to observe the early development of the flames and the temperature of the liners and is positioned at 1/6th of the chambers ($x_{{}_{RIBBED}}=30$ mm, $x_{{}_{SMOOTH}}=25$ mm). The two other cuts are used to observe the high temperature spots observed at 1/3rd of the chambers ($x_{{}_{RIBBED}}=90$ mm, $x_{{}_{SMOOTH}}=75$ mm) and 2/3rd of the chambers ($x_{{}_{RIBBED}}=120$ mm, $x_{{}_{SMOOTH}}=100$ mm). The penetration of hot gas in the liner is visualized with the three cuts in the two chambers Figure 16. The solid liner is impacted by the presence of the ribs, and strong temperature gradients are observed between the top of the ribs (in contact with hot gases) and the bottom of the ribs (in contact with fresh gases). The most critical part for design of the ribbed liner is located at the top of the ribs, where the temperature is the highest. This indicates that the design of thrust chambers should pay attention to the hot gas side wall temperature in the ribbed cylinder.

**Figure 17:**
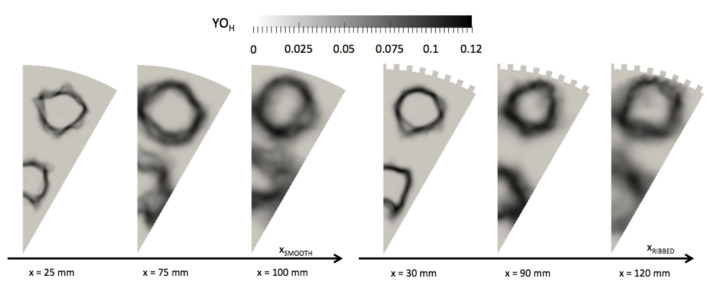
Time-averaged OH mass fraction on cross-section cuts for the smooth and ribbed configurations.

Maximum temperatures in the burnt gases are of the same order in both configurations, and flame diameters along the chamber are similar. High-temperature burnt gases flow closer to the wall in the ribbed configuration. The cross-section cuts of OH mass fraction give the position of the flame in the smooth and in the ribbed configurations Figure 17. The flame is much closer to the wall in the ribbed chamber than in the smooth chamber. The proximity of the flame is not due to the higher heat flux extraction capacity obtained with the ribs (increase of exchange surface about 85%), which should push back the flame from the wall. The proximity of the flame is attributed to the penetration of the bulk flow in the micro-channels due to lower axial velocity. The presence of the ribs then affects the curvature of the flame as seen at x=120 mm in the ribbed chamber where the $Y_{OH}$ field is wrinkled around the top of the ribs. On Figure 17, one can observe that at the axial position x=100 mm in the smooth chamber, the flame diameter decreases, announcing the end of the flame. In the ribbed chamber at the equivalent axial position x=120 mm, the flame diameter is still large, the ribbed chamber is longer, and the flame closes later in the chamber.

#### 4.3.1. Stratification in the Inter-Rib Region

The channel formed by the inter-rib regions shows an important stratification in temperature. In the downstream part of the chamber, temperature of burnt gases seen by the top of the ribs is T≈1800 K, while temperature seen by the bottom of the ribs is T≈600 K.

**Figure 18:**
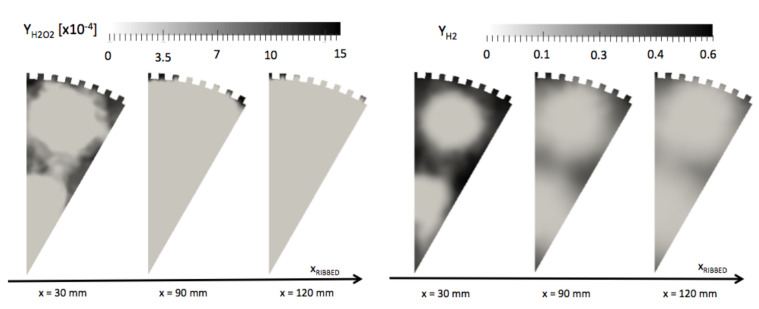
Time-averaged $H_{2}$ and $H_{2}O_{2}$ mass fractions on cross-sections cuts for the smooth and ribbed configurations.

**Figure 19:**
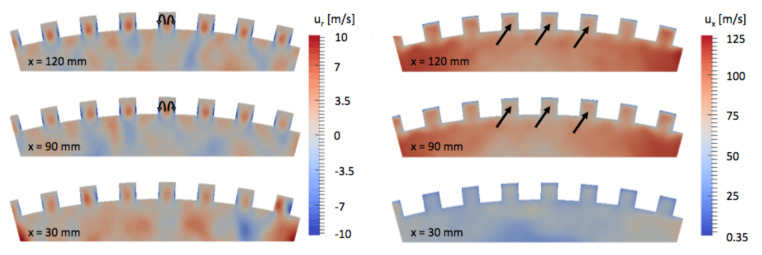
Time-averaged fields of radial and axial velocity $u_{r}$ and $u_{x}$ on cross-section cuts on the ribbed combustion chamber.

The stratification not concerns only temperature; the composition of the mixture in the vicinity of the ribs also changes. Unburnt gases $H_{2}$ and radicals such as $H_{2}O_{2}$, formed early in the lateral recirculation zones, are present in higher concentration in the inter-rib channels (Figure 18). This effect is not due to chemistry as the flame does not penetrates the inter-rib space. The presence of these fresh gases in the inter-rib region is due to a suction effect induced by the ribbed geometry. In Figure 19, time-averaged fields of the axial $u_{x}$ and radial $u_{r}$ components of the velocity are represented on cross-section cuts. Lower velocities are observed in the micro-channels. The hot gases penetrate the micro-channels under a suction effect induced by the velocity gradient. The radial component of the velocity $u_{r}$ confirms a structure of two contra-rotative vortices, which establish themselves all along the chamber and act like fresh gases at the beginning of the chamber. The fresh gases are captured in the micro-channels and create a film cooling while feeding the flame. Fresh gases stuck in the channels of the ribbed wall lower the efficiency of the ribs. As a result, doubling the exchange surface does not result in doubling the heat transfer evacuated.

#### 4.3.2. Heat Transfer

**Figure 20:**
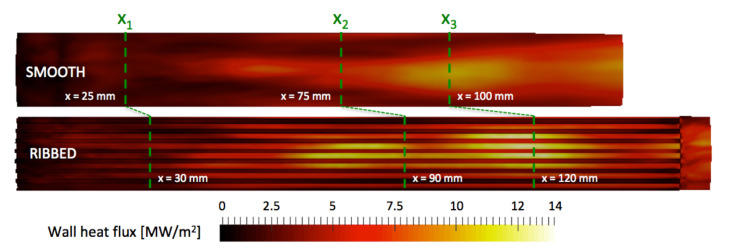
Time-averaged wall heat flux of the $30^{\circ }$ sector simulation of smooth (**top**) and ribbed (**bottom**) burners.

The distributions of wall heat fluxes for the two configurations are represented in Figure 20. The organization is similar to the temperature distribution with regions of high fluxes close to the high temperature spots evidenced previously.

**Figure 21:**
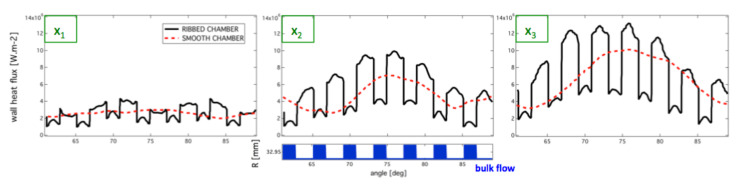
Time-averaged of wall heat flux circumferential for the ribbed and smooth configurations at three axial positions. The distance from the axis of the chamber to the wall position *R* is given to identify ribs and inter-rib regions.

Time-averaged wall heat flux circumferential distributions are plotted for the three axial position (1/6th, 1/3rd, and 2/3rd) on Figure 21. The distance of the wall to the chamber axis *R* is given to visualize the rib location. The heat transfer computed in the near injector region is low due to fresh mix of unburnt hydrogen and burnt gases’ lateral recirculation. The wall heat flux seen by the top of the ribs is always higher than the heat flux seen by the bottom of the inter-rib region. On the sides of the ribs, the wall heat flux passes from the top-rib high values to the bottom-rib lower value. Interestingly, the bases and top face of the ribs show more intense heat-fluxes than the corners due to higher skin friction in these regions as expected from the wall-modeled LES of ribbed channel flows presented in [43]. Smooth wall combustion chamber exhibits intermediate values of wall heat flux, between the top-rib values and the bottom-rib values of the ribbed configuration. The increase of the surface exchange is responsible for heat transfer enhancement brought about by the ribbed configuration.

### 4.4. Heat Transfer: Comparison with Experiment

**Figure 22:**
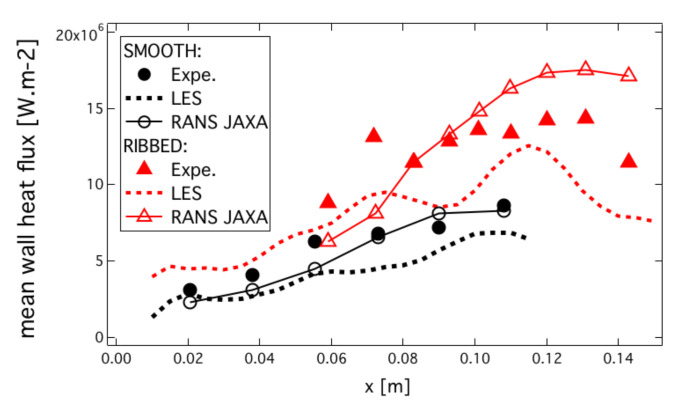
Time-averaged wall heat flux of the ribbed and smooth configuration obtained by the present CHT-LES confronted to the experiment. RANS calculation published by JAXA [11] are also represented.

In this section, heat fluxes obtained with the CHT-LES for both configurations are compared to heat transfer measurements from the experiments as well as with RANS results published by JAXA [11]. Time-averaged wall heat fluxes of ribbed and smooth configurations are plotted along the axial direction of the combustion chamber on Figure 22. For the ribbed configuration, time-averaged wall heat flux $q_{w,r}$ has been integrated along the wet perimeter (following the wall abscissa) for different axial positions in the chamber and scaled by the projected wet perimeter (in the present case the smooth chamber wet perimeter) following:

(8) $$q_{w,r}^{s}\left(x\right)=\frac{1}{P_{s}}\int_{P_{r}}q_{w,r}\left(x\right)dl$$

where $P_{s}$ and $P_{r}$ are the respective perimeters of the smooth and ribbed chambers. The global evolution of the heat flux along the axis is recovered in both simulations, and fluxes are under-estimated by ≈10–20% compared to experimental measurements. RANS-published simulation [11] was conducted with a droplet injection model fitted to match heat flux measurement for the smooth chamber. However, the results of the ribbed configuration show under-predicted heat flux in the middle of the chamber and over-predicted heat-flux at the end.

In the present coupled applications, the outer wall of the liner temperature was imposed to the cooling water temperature. The geometry of the liner on the coolant side has also been simplified. Taking account of the real geometry and imposing exchange coefficient boundary condition on the coolant side of the liner would improve the prediction of the coupled simulation. Nonetheless, the general tendencies of the predicted heat fluxes along the chamber wall are well predicted by the numerical simulation, as is the improvement in heat transfer between the smooth and ribbed walls.

The rib efficiency defined by:

(9) $$\eta_{r}=\frac{q_{w,r}/q_{w,s}}{A_{r}/A_{s}}$$

with $q_{w,r}$ and $q_{w,s}$ the integrated wall heat fluxes for the ribbed and smooth configuration, and $A_{r}$ and $A_{s}$ the surface of the ribbed and smooth wall, is estimated to $\eta_{r}=0.795$. This means that almost 80% of additional heat flux is extracted thanks to the ribbed liner for the same mean chamber diameter as often reported in the literature [3,10,11,12].

## 5. Conclusions

Heat transfer is a key design point for rocket engines, and maximizing the heat flux extracted from the chamber can lead to serious gain for the cooling and feeding systems, especially when it comes to expander-type feeding cycles where the thermal energy absorbed by the coolant is converted into kinetic energy to drive the turbo-pumps of the feeding system. To this end, a technical solution is to design longitudinally ribbed combustor walls. This solution is explored in a dual experiment from JAXA, operated at JAXA/Kakuda space center, which comprises two equivalent chambers, fired at the same operating points, the first with smooth walls and the second with ribs. The methodology proposed in this work to numerically predict the flame topology and associated heat fluxes is based on high-fidelity Large Eddy Simulation Conjugate Heat Transfer along with a reduced kinetic mechanism for the prediction of $H_{2}/O_{2}$ chemistry, liquid injection model *LOx* sprays, and the use of a specific wall modeling to correctly predict heat flux for the large temperature ratio between the bulk flow and the chamber walls. Conjugate Heat Transfer allows one to set the unknown thermal boundary condition further away from the investigated region of experimental measurements: imposing temperature at the outer boundary of the solid liner in contact with the coolant is easier than imposing the boundary condition directly on the fluid domain where a temperature mapping of the ribbed or smooth surface is required. The coupling strategy ensures a rapid convergence of the CHT problem for a limited additional cost compared to a fluid only simulation. The comparison with experiments is conclusive with a slight under-estimation of the wall heat flux observed. The heat transfer enhancement of the ribbed configuration with respect to the smooth walls is coherent with what has been observed in the literature, with an increase of around +80% of wall heat flux extracted for the same chamber diameter. 

## Figures and Tables

**Table 1:** Geometrical characteristics of the two JAXA calorimeter chambers [11].

	Ribbed Case	Smooth Case
chamber length [mm]	153	117
chamber diameter [mm]	65/67	66
number of injectors	18	18

**Table 2:** Operating conditions of the two JAXA calorimeter chambers [11].

	Ribbed	Smooth
$O_{2}/H_{2}$ mass ratio	5.6	5.3
chamber pressure [bar]	35	36
total mass flow ($H_{2}+O_{2}$) [kg·m${}^{-3}$]	0.616	0.626
$O_{2}$ injection temperature [K]	95	95
$H_{2}$ injection temperature [K]	275	275

**Table 3:** Liquid injection characteristics for the ribbed and smooth JAXA calorimeter chambers.

	Ribbed	Smooth
We	14,485	14,500
$Re_{l}$	92,000	90,700
*J*	1.8	1.85
cone length *L* [mm]	10.6	10.85
injection diameter μ [m]	41.2	40.7
$\theta_{lip}$	$38.62^{\circ }$	$38.54^{\circ }$
$\alpha_{l}$	0.118	0.12
St	0.76	0.11

**Table 4:** Material properties of the solid parts.

	Copper Alloy	Inconel600
density $\rho_{s}$	8814 kg/m${}^{3}$	8470 kg/m${}^{3}$
heat capacity $C_{s}$ at 300 K	377 J/kg/K	444 J/kg/K
conductivity $\lambda_{s}$ at 300 K	322 W/m/K	14.9 W/m/K

**Table 5:** Mesh size employed for JAXA chambers coupled computations.

	Ribbed Chamber	Smooth Chamber
Fluid mesh	30.6M.cells	31.5M.cells
Solid mesh	25M.cells	8M.cells

## Data Availability

Not applicable.
